# Kniegelenknahe Osteotomien: operative Planung mithilfe von CT-3-D-Analyse, patientenspezifischen Schnitt- und Korrekturblöcken

**DOI:** 10.1007/s00064-023-00814-w

**Published:** 2023-06-14

**Authors:** Lazaros Vlachopoulos, Sandro F. Fucentese

**Affiliations:** Klinik für Orthopädie, Universitätsklinik Balgrist, Universität Zürich, Forchstr. 340, 8008 Zürich, Schweiz

**Keywords:** Osteotomie, Tibia, Femur, Patientenspezifische Instrumente, 3‑D computerassistierte orthopädische Chirurgie, Osteotomy, Tibia, Femur, Patient-specific instruments, Three-dimensional computer-assisted orthopaedic surgery

## Abstract

**Operationsziel:**

Ziel ist, durch eine Osteotomie entweder die prätraumatischen anatomischen Verhältnisse wiederherzustellen oder die Belastung in weniger betroffene Kompartimente zu verlagern.

**Indikationen:**

Die Indikation für computerassistierte 3‑D-Analyse und die Verwendung von patientenspezifischen Schnitt- und Korrekturschnittblöcken ist neben „einfachen“ Deformitäten insbesondere auch bei multidimensionalen komplexen (v. a. posttraumatischen) Deformitäten gegeben.

**Kontraindikationen:**

Allgemeine Kontraindikationen für die Durchführung einer Computertomographie (CT) oder für einen offenen Zugang für die Durchführung der Operation.

**Operationstechnik:**

Anhand CT-Untersuchungen der betroffenen und ggf. der kontralateralen gesunden Extremität als gesunde Vorlage (einschließlich Hüft‑, Knie- und Sprunggelenk) werden 3‑D-Computer-Modelle erzeugt, welche für die 3‑D-Analyse des Ausmaßes der Deformität als auch für die Berechnung der Korrekturparameter verwendet werden. Für die exakte und vereinfachte intraoperative Umsetzung des präoperativen Plans werden individualisierte Schablonen für die Osteotomie als auch die Reposition mittels 3‑D-Druck hergestellt.

**Weiterbehandlung:**

Teilbelastung an Unterarmstützen ab dem ersten postoperativen Tag. Belastungsaufbau abhängig von den Röntgenkontrollen nach 6 Wochen. Keine Limitierung des Bewegungsumfangs.

**Ergebnisse:**

Es gibt mehrere Studien, welche die Genauigkeit der Umsetzung der geplanten Korrektur für kniegelenknahe Korrekturosteotomien mit der Verwendung patientenspezifischer Schnitt- und Korrekturschnittblöcke analysiert haben mit vielversprechenden Ergebnissen.

## Abstract (en)

**Objective:**

The goal of osteotomy is either to restore pretraumatic anatomic conditions or to shift the load to less affected compartments.

**Indications:**

Indications for computer-assisted 3D analysis and the use of patient-specific osteotomy and reduction guides include “simple” deformities and, in particular, multidimensional complex (especially posttraumatic) deformities.

**Contraindications:**

General contraindications for performing a computed tomography (CT) scan or for an open approach for performing the surgery.

**Surgical technique:**

Based on CT examinations of the affected and, if necessary, the contralateral healthy extremity as a healthy template (including hip, knee, and ankle joints), 3D computer models are generated, which are used for 3D analysis of the deformity as well as for calculation of the correction parameters. For the exact and simplified intraoperative implementation of the preoperative plan, individualized guides for the osteotomy and the reduction are produced by 3D printing.

**Postoperative management:**

Partial weight-bearing from the first postoperative day. Increasing load after the first x‑ray control 6 weeks postoperatively. No limitation of the range of motion.

**Results:**

There are several studies that have analyzed the accuracy of the implementation of the planned correction for corrective osteotomies around the knee joint with the use of patient-specific instruments with promising results.

## Vorbemerkungen

Kniegelenknahe Osteotomien haben in den letzten Jahrzehnten zunehmend Beachtung gefunden für die Behandlung von unikompartimentellen Gonarthrosen bei jüngeren Patienten als auch für die Behandlung bzw. Behebung prädisponierender Faktoren bei ligamentären Instabilitäten.

Achsabweichungen in der koronaren Ebene (Varus‑/Valgusdeformitäten) sind dabei der wahrscheinlich häufigste Grund für eine kniegelenknahe Osteotomie, welche mittels einer proximalen Tibiaosteotomie oder distalen Femurosteotomie adressiert werden können.

Darüber hinaus finden Korrekturen in der sagittalen Ebene, z. B. Slope-korrigierende Tibiakopfosteotomien, bei sagittalen Instabilitäten zunehmende Beachtung, ebenso wie Korrekturen in der transversalen Ebene bei patellofemoralen Pathologien, z. B. derotierende Femurosteotomien. Während bei Letzteren für die Quantifizierung der Deformität zumindest eine Messung anhand einer 3‑D-Schichtbildgebung notwendig ist, ist der aktuelle Standard für eine Korrekturosteotomie weiterhin die 2‑dimensionale (2-D-)Analyse gemäß den Prinzipien von Paley [1]. Die präoperative 3‑dimensionale (3-D-)Planung von Osteotomien der unteren Extremität hat jedoch angesichts der modernen chirurgischen Technologien zunehmend an Bedeutung gewonnen [2].

Für eine präoperative 3‑D-Analyse einer Deformität werden CT-Daten der betroffenen Extremität benötigt, welche das Hüftzentrum, proximales Femur, das Kniezentrum mit distalem Femur, proximaler Tibia und proximaler Fibula sowie das obere Sprunggelenkzentrum mit distaler Tibia, distaler Fibula und Talus beinhalten. Während die CT-Auflösung im Bereich der geplanten Osteotomie möglichst hoch gewählt sein sollte (Schichtdicke ca. 1 mm), kann diese für kniegelenknahe Osteotomien im Bereich des distalen Unterschenkels und insbesondere auch im Bereich der strahlensensibleren Regionen um das Becken niedriger gewählt werden, um die Strahlenbelastung zu reduzieren [3]. Aus gleichen Überlegungen können zusätzlich die Schaftbereiche des Femurs und des Unterschenkels ausgespart werden.

**Figure 1:**
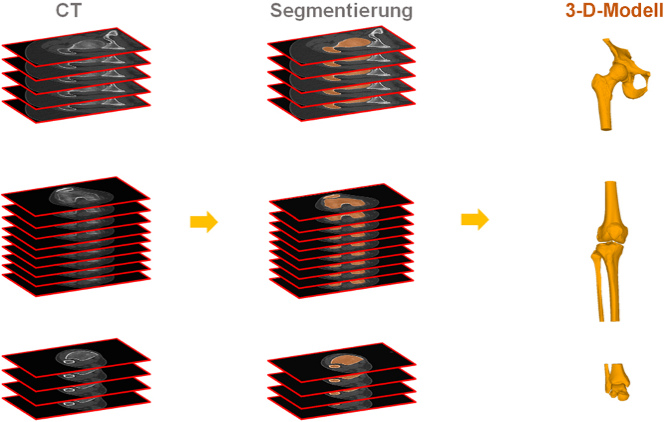

Der grundlegende Schritt für die 3‑D-Analyse ist die Generierung von 3‑D-Oberflächenmodellen der pathologischen Extremität und ggf. auch der kontralateralen gesunden Extremität [4, 5]. Dabei werden häufig Thresholding- und Region-growing-Algorithmen für die Segmentierung (Abb. 1) verwendet und der Marching-cubes-Algorithmus für die Generierung des 3‑D-Modells (Abb. 1; [5]).

Da die meisten Messungen und auch die geplanten Korrekturen auf 2‑dimensional (2-D) projizierten Messwerten basieren, ist die a.-p-Ausrichtung der Extremität von essenzieller Bedeutung. Dabei wird ähnlich wie beim Erstellen eines Orthoradiogramms, bei dem darauf geachtet wird, dass die Patella nach ventral gerichtet ist, die Patella als Referenz für die Ausrichtung der Extremität verwendet [4, 6]. Dies ist insbesondere bei größeren Deformitäten sowie Flexionskontrakturen des Kniegelenks von Bedeutung [7].

**Figure 2:**
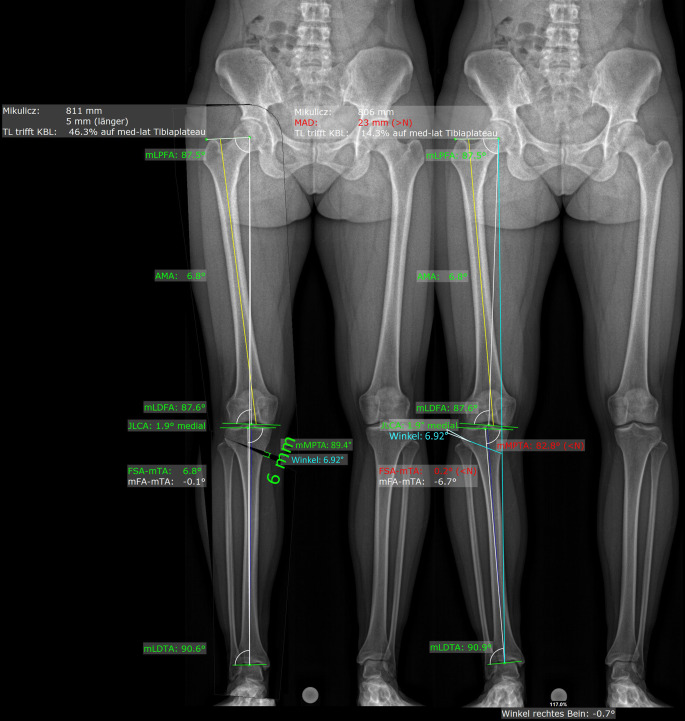

**Figure 3:**
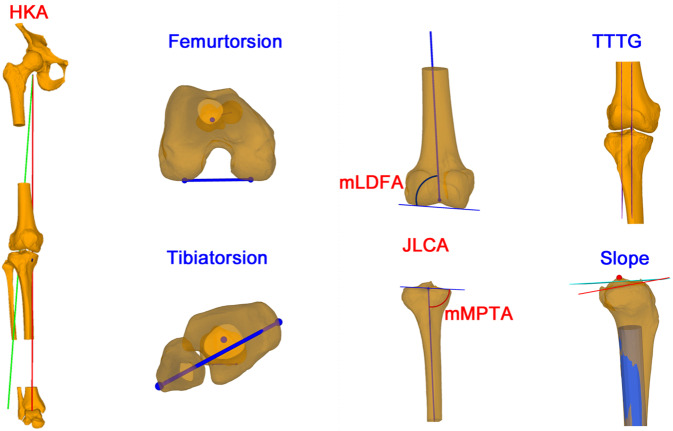

Die Analyse einer Deformität kann dabei den Prinzipien von Paley folgen ([1]; Abb. 2), wobei standardisierte Landmarken in 3‑D verwendet werden für die Berechnung der projizierten Messwerte [6, 8]. Für diese landmarkenbasierte Deformitätsanalyse werden v. a. folgende Messwerte definiert, welche bei der Bestimmung des Korrekturausmaßes als auch des Korrekturortes (z. B. distaler Femur vs. proximale Tibia) herangezogen werden können (Abb. 3).

- HKA mechanische femorotibiale Winkel („hip knee ankle angle“)
- mLDFW (mechanischer lateraler distaler Femurwinkel)
- mMPTW (mechanischer medialer proximaler Tibiawinkel)
- JLCA (Gelenklinienwinkel „joint-line conversion angle“)
- Femorale Torsion
- Tibiale Torsion
- Tibialer Slope
- TTTG („tibial tuberosity trochlear groove“)

Die Belastung der Extremität hat dabei einen großen Einfluss insbesondere auf den HKA als auch auf den JLCA [8]. Dies muss insbesondere bei der Bestimmung des Korrekturwinkels berücksichtigt werden. Ein möglicher Lösungsansatz zur Berücksichtigung der Belastung bei der Verwendung von CT-Daten ist entweder die Verwendung einer belasteten CT-Untersuchung (Weight-bearing-CT) oder die 3‑D-2-D-Regristiereung, d. h. die Fusion von unbelasteten 3‑D-CT-Daten mit belasteten 2‑D-Orthoradiogrammen [2].

**Figure 4:**
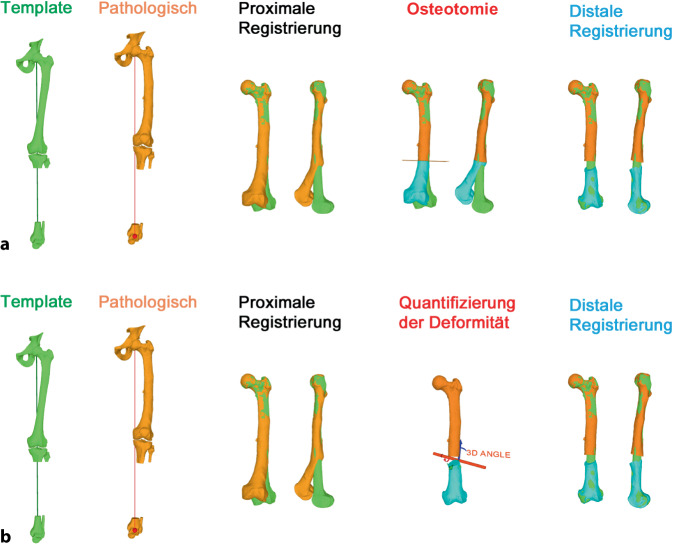

Eine weitere Möglichkeit einer Deformitätsanalyse neben der landmarkenbasierten Methode ist die templatebasierte Methode, welche insbesondere bei posttraumatischen, multidimensionalen Deformitäten von Vorteil ist (Abb. 4). Dabei kann entweder die gespiegelte kontralaterale Anatomie [9, 10] oder ein statistisches Modell [10, 11] als Vorlage verwendet werden. Durch das Übereinanderlegen der 3‑D-Modelle der pathologischen Extremität und des Templates proximal der Deformität kann das Ausmaß der Deformität bereits visualisiert werden (Abb. 4 proximale Registrierung). Nach einer simulierten Osteotomie auf Höhe der Deformität wird anschließend das 3‑D-Modell der pathologischen Extremität distal der Deformität mit dem Template übereinandergelegt (Abb. 4 distale Registrierung). Dieser Unterschied zwischen proximaler und distaler Registrierung entspricht dem Ausmaß der Deformität und kann als Transformationsmatrix kodiert und für die weitere 3‑D-Planung verwendet werden, z. B. Berechnung einer Rotationsachse oder einer Single-Cut-Osteotomie-Ebene. Das Ergebnis einer templatebasierten Deformitätsanalyse muss jedoch immer mit der vorliegenden Pathologie und auch der Klinik abgeglichen werden, da physiologisch vorkommende intraindividuelle bilaterale Unterschiede das Ausmaß der tatsächlich vorliegenden Deformität verfälschen können [12].

In den letzten Jahren gibt es zunehmende Studien, welche den Vorteil von 3‑D-Planung und patientenspezifischen Instrumenten (PSI) bei distalen Femurosteotomien und proximalen Tibiaosteotomien belegen und insbesondere die hohe Genauigkeit der Korrektur und den geringen Anteil an Outliern hervorheben [13–17]. Randomisierte Studien fehlen jedoch, und ebenso fehlt auch der Vergleich zur klassischen Navigation.

**Figure 5:**
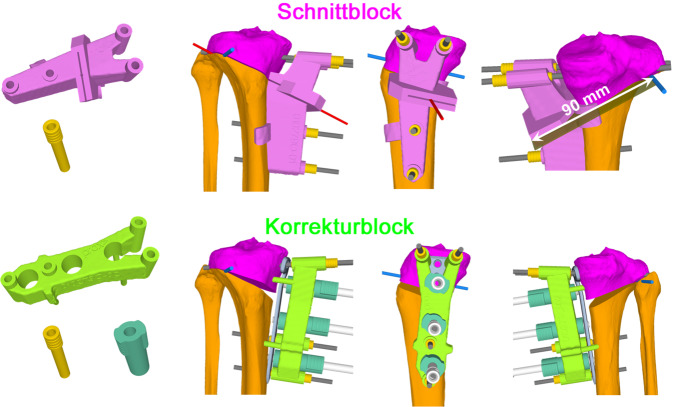

Im folgenden Artikel werden die Grundprinzipien der Umsetzung kniegelenknaher Osteotomien anhand PSI beschrieben (Abb. 5 und 6), wobei das Hauptaugenmerk auf Korrekturen in der koronaren als auch der axialen Ebene gelegt wird. Mit zunehmender Erfahrung anhand dieser „einfacheren“ Korrekturosteotomien können die Prinzipien auch für „komplexere mehrdimensionale“ Korrekturosteotomien angewendet werden. Die Kombination einer Korrektur in mehreren Ebenen, z. B. koronaren und sagittalen Ebene, kann durch eine einzelne Rotation um eine kombinierte Rotationsachse erreicht werden (Abb. 4b, rote Rotationsachse). Die intraoperative Umsetzung unter Berücksichtigung dieser resultierenden Rotationsachse ist jedoch ohne PSI kaum möglich, wobei aus diesem Grund die Anwendung von PSI insbesondere bei mehrdimensionalen posttraumatischen Deformitäten nicht mehr wegzudenken ist.

**Figure 6:**
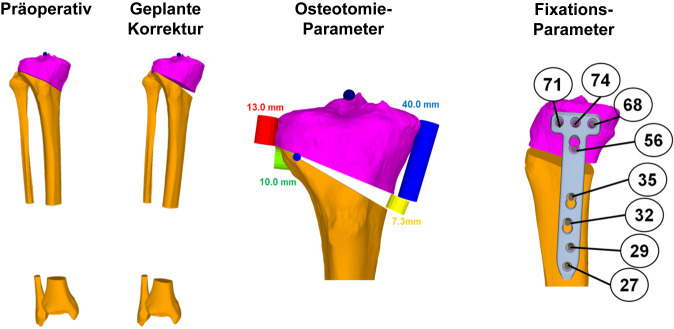

## Operationsprinzip und -ziel

Ziel ist, durch eine Osteotomie entweder die prätraumatischen anatomischen Verhältnisse wiederherzustellen oder die Belastung in weniger betroffene Kompartimente zu verlagern. Patientenspezifische Instrumente (PSI) ermöglichen dabei die exakte intraoperative Umsetzung des präoperativen Planes. Bei der beschriebenen Technik werden jeweils 2 Schanz-Schrauben proximal bzw. distal der geplanten Osteotomie verwendet, wobei PSI insbesondere für folgende Schritte verwendet werden: zum Platzieren der Schanz-Schrauben, für die Osteotomie und auch für die Korrektur unter Verwendung der Schanz-Schrauben.

## Vorteile

- Höhere Genauigkeit
- Weniger Outlier
- Reduktion der Operationszeit
- Reduktion der Verwendung der intraoperativen Fluoroskopie

## Nachteile

- Kosten
- Herstellungszeit für die PSI
- Weniger intraoperative Flexibilität
- Kein minimal-invasiver Zugang

## Indikationen

- Einfache und komplexe kniegelenknahe Korrekturosteotomien

## Kontraindikationen

- Allgemeine Kontraindikationen für die Durchführung einer Computertomographie (CT) oder für einen offenen Zugang für die Durchführung der Operation wie Infekt, schlechte Weichteile.

## Patientenaufklärung

- Allgemeine Risiken einschließlich Gefäß‑, Nervenverletzungen und Infektion
- Kompartmentsyndrom
- Über- und Unterkorrektur
- Pseudarthrose

## Operationsvorbereitungen

- Rx a.-p./seitlich und Ganzbeinaufnahme (Orthoradiogramm)
- CT der betroffenen Extremität mit einer Schichtdicke von 1 mm einschließlich Hüftzentrum, proximalem Femur, Kniezentrum mit distalem Femur, proximaler Tibia und proximaler Fibula sowie das obere Sprunggelenkzentrum mit distaler Tibia, distaler Fibula und Talus
- 3‑D-Planung und Herstellung patientenspezifischer Instrumente

## Instrumentarium

- Patientenspezifische Instrumente (Schnitt- und Korrekturblöcke, Bohrhülsen; MyOsteotomy, Medacta SA, Schweiz)
- Eva-Haken
- Langenbeck-Haken
- 4 × 4,0 mm Schanz-Schrauben
- 2,0-mm-K-Draht
- Oszillierende Säge mit einem breiten Sägeblatt (Länge 90 mm, Dicke 1 mm) und einem schmalen Sägeblatt (Länge 50 mm, Dicke 1 mm) für die biplanare Osteotomie
- Osteotomiemeißel
- Knochenspreitzer
- 3,5-mm-Kortikalisschrauben
- Osteosynthese‑/Osteotomieplatte, welche bei der präoperativen 3‑D-Planung als optimal definiert wurde
- Bildwandler

## Anästhesie und Lagerung

- Allgemein- oder Regionalanästhesie
- Rückenlagerung auf röntgendurchlässigem Tisch
- Ev. Beinhalter
- Ev. Blutsperre

## Operationstechnik

**Figure 7:**
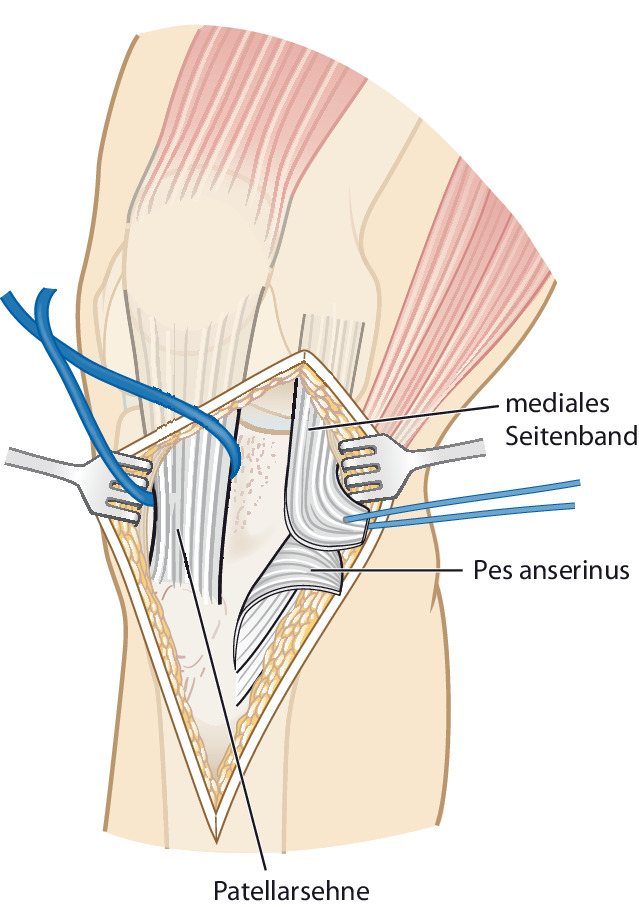

**Figure 8:**
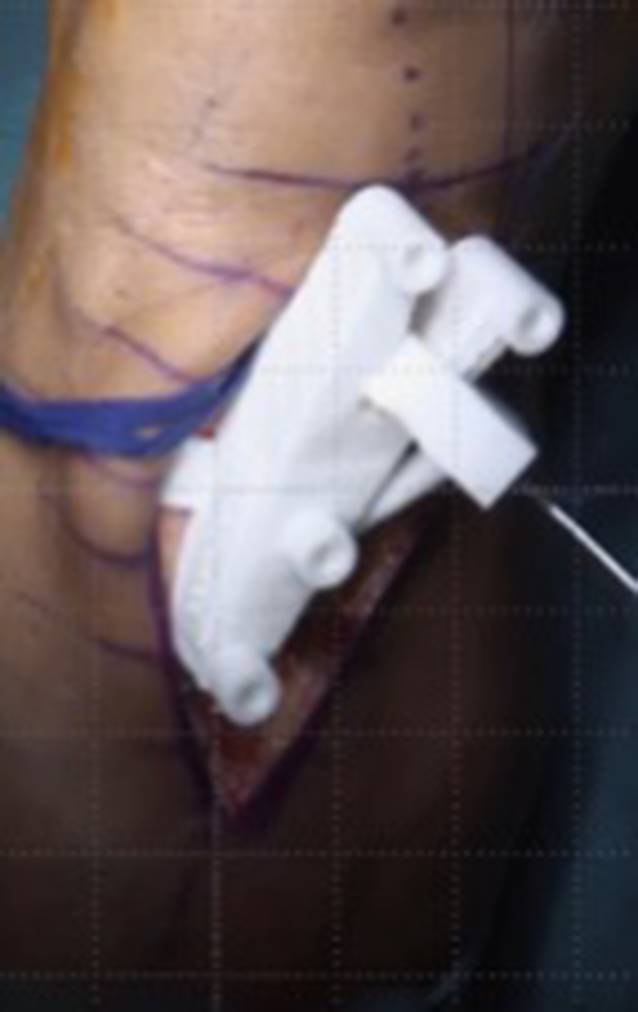

**Figure 9:**
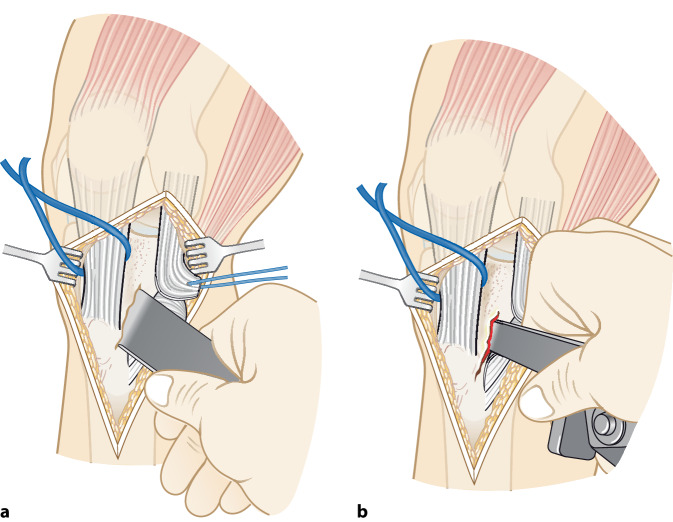

**Figure 10:**
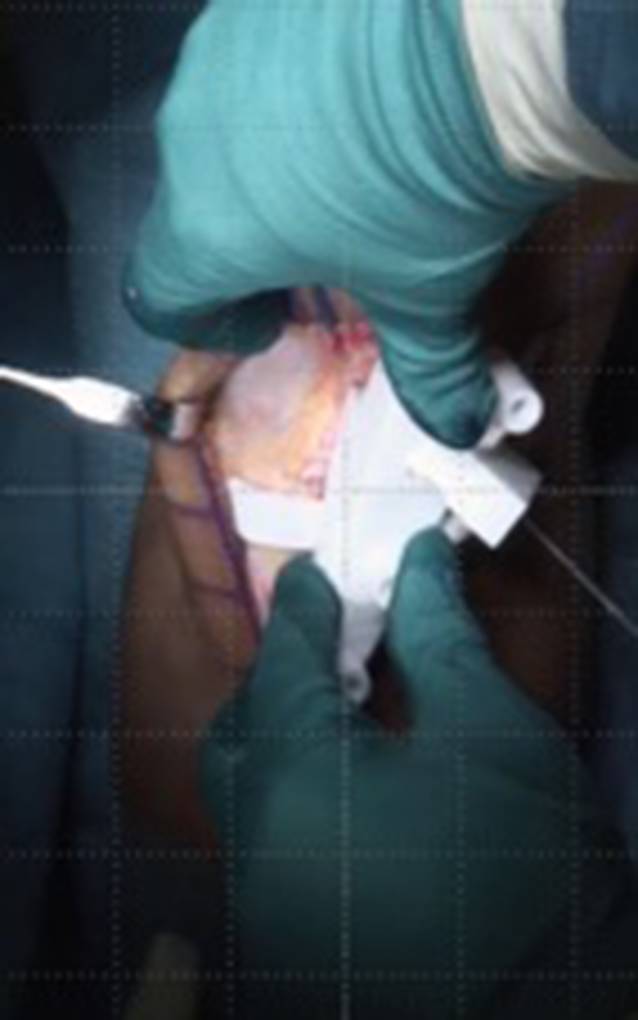

**Figure 11:**
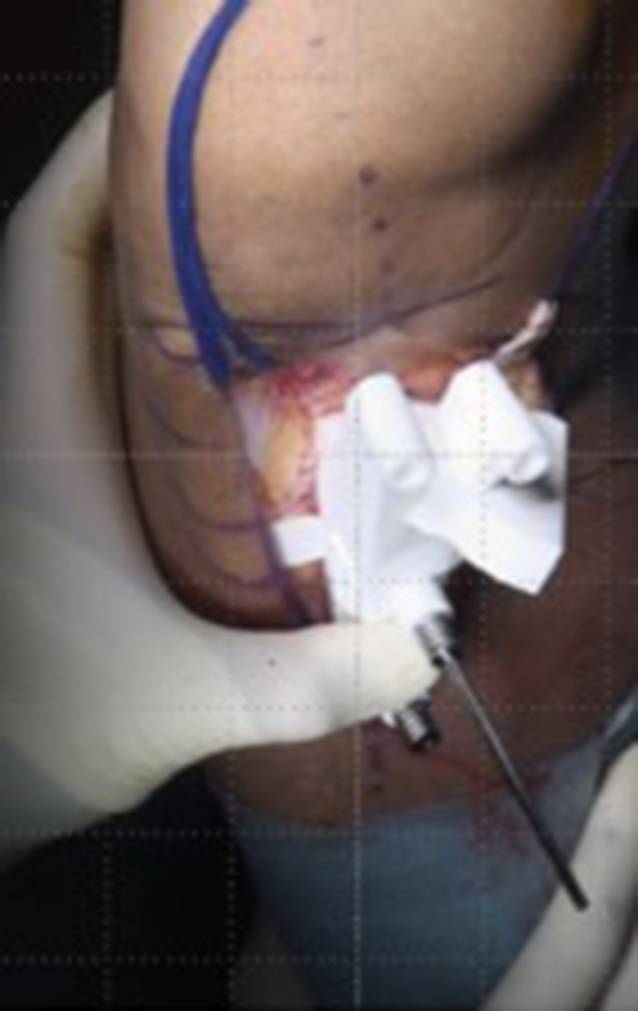

**Figure 12:**
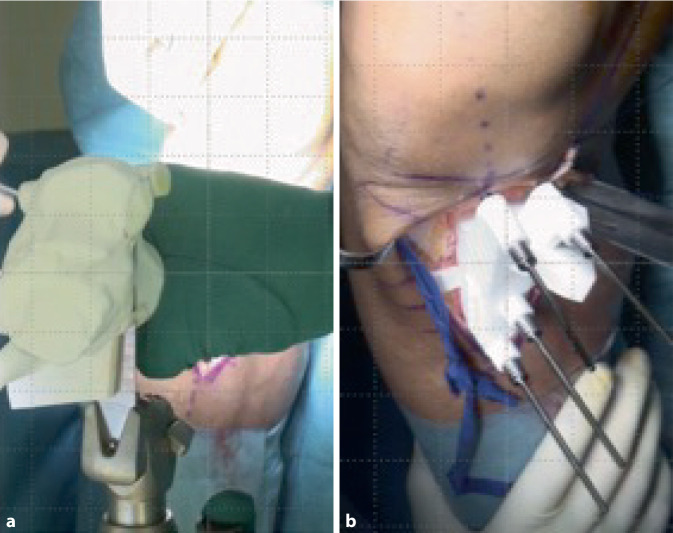

**Figure 13:**
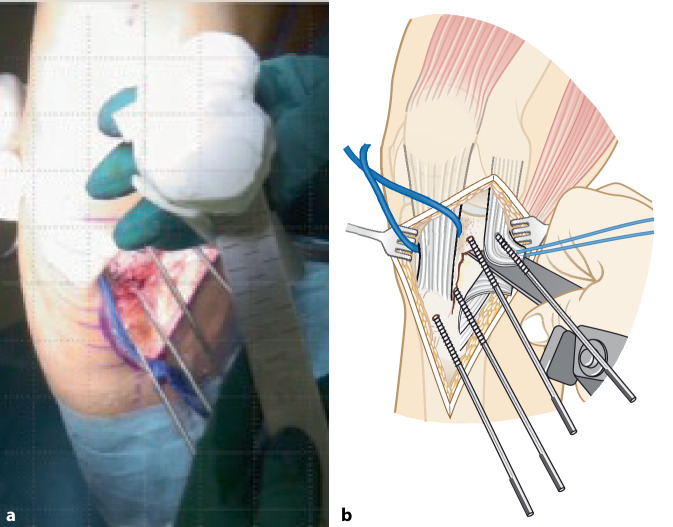

(Abb. 7, 8, 9, 10, 11, 12, 13, 14** und **15)

**Figure 14:**
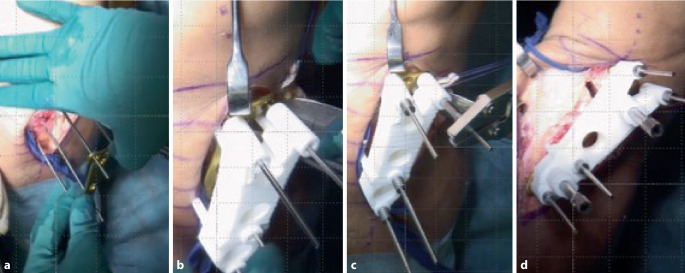

**Figure 15:**
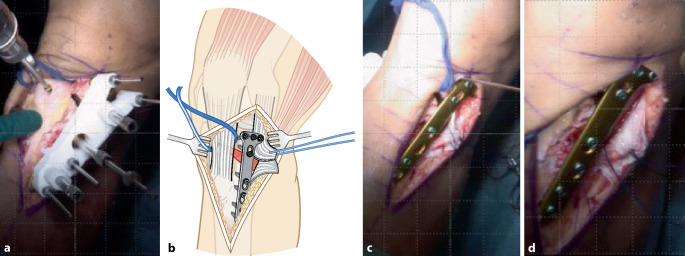

## Postoperative Behandlung

- Elastische Wickelung des Beins inklusive des Oberschenkels im Operationssaal
- Physiotherapie ab dem 1. postoperativen Tag
- Teilbelastung für 6 Wochen
- Thromboseprophylaxe z. B. mit niedermolekularen Heparinen bis zum Erreichen der sicheren Vollbelastung

## Fehler, Gefahren, Komplikationen

- Über- bzw. Unterkorrektur durch ungenügende oder falsche präoperative Planung und nicht korrekte Platzierung des Schnittblockes bzw. nicht stabile Reposition mit dem Korrekturblock aufgrund großer Spannungsverhältnisse. Durch eine nicht korrekte Rotation des Schnittblocks wird ein Fehler in einer zweiten Ebene eingebaut werden, wobei die Korrektur in der primär geplanten Ebene im Normalfall eher zu einer Unterkorrektur führen wird
- Fraktur durch die Schanz-Schrauben-Löcher bei Platzierung neben der Platte
- Hinge-Fraktur mit möglichem Korrekturverlust und Delayed-Union bzw. Non-Union

## Ergebnisse

**Figure 16:**
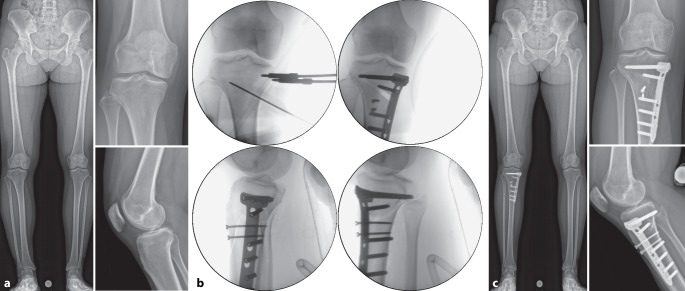

**Figure 17:**
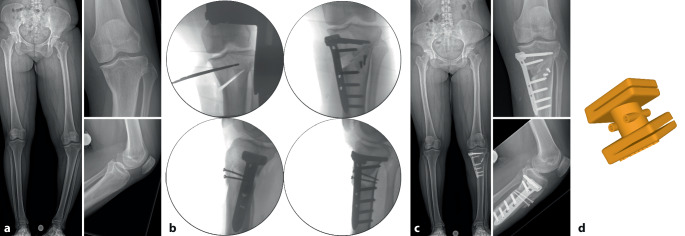

**Figure 18:**
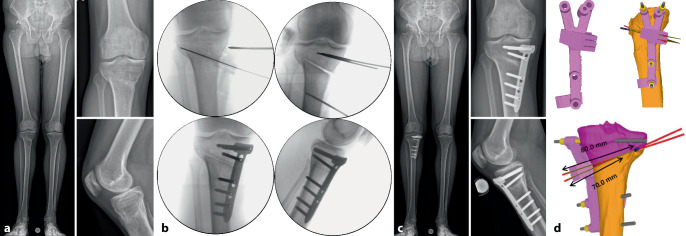

**Figure 19:**
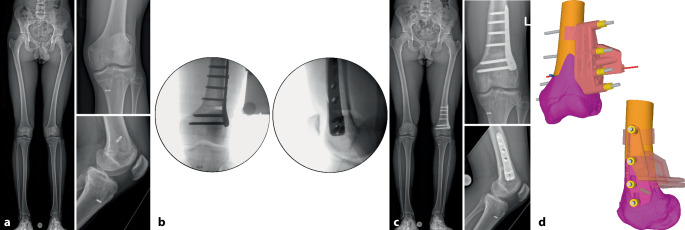

Es gibt mehrere Studien, welche die Genauigkeit der Umsetzung der geplanten Korrektur für kniegelenknahe Korrekturosteotomien mit der Verwendung patientenspezifischer Schnitt- und Korrekturschnittblöcke analysiert haben mit vielversprechenden Ergebnissen [13, 15, 17, 22–24]. Der klinische Benefit der gewonnenen Genauigkeit ist jedoch noch unklar. Im Folgenden werden klinische Beispiele von kniegelenknahen Korrekturosteotomien illustriert mit Hauptaugenmerk auf die Besonderheiten in den präoperativen Planung bzw. der intraoperativen Umsetzung (Abb. 16, 17, 18, 19, 20 und 21).

**Figure 20:**
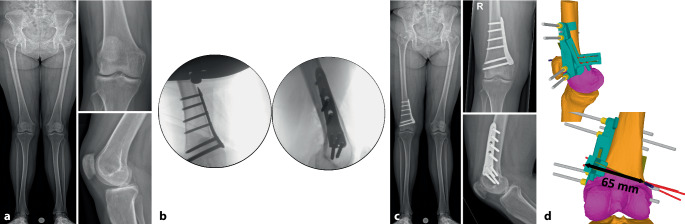

**Figure 21:**
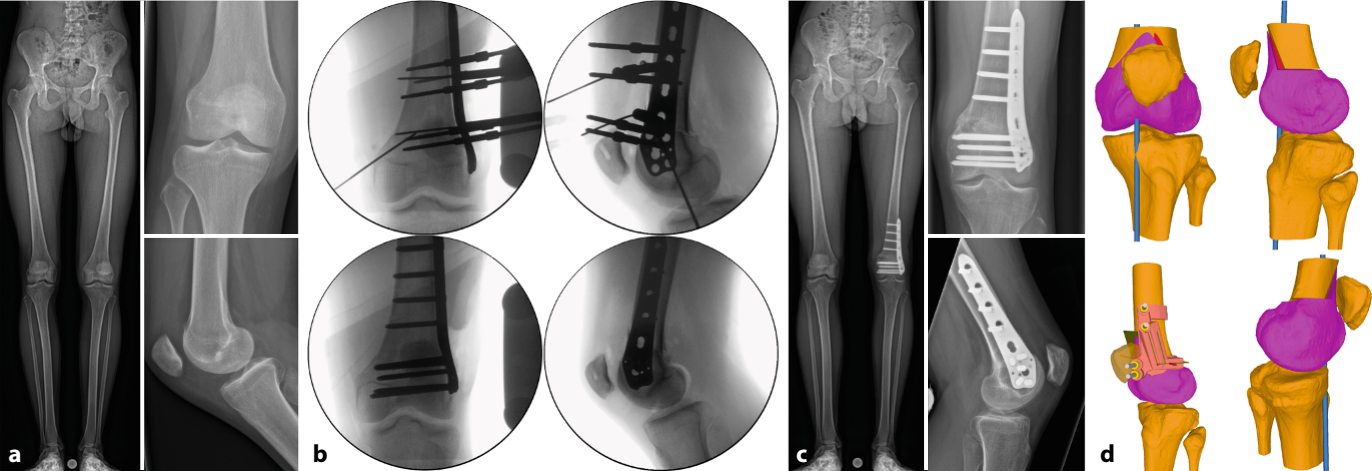

Prinzipiell besteht die Möglichkeit, die Schanz-Schrauben durch die Platte oder neben der Platte zu platzieren. Der Hauptgrund, die Schraubenlöcher zu verwenden, ist, dass hierdurch der Guide und der Zugang etwas kleiner gewählt werden können. Die Platzierung der Platte am Femur ist jedoch am Computer am 3‑D-Modell etwas schwieriger, insbesondere da bisher keine Weichteile in der Planung berücksichtigt werden. So kann z. B. das distale Plattenende den Tractus iliotibialis bei einem lateralen Zugang irritieren. Aus diesem Grund bevorzugen wir, die Platte am Femur neben den Pins zu platzieren, damit wir hier etwas mehr variieren können. Das Ausmaß der Korrektur wird hierdurch jedoch voraussichtlich nicht verändert, und man kann sich für die Platzierung der Platte dennoch an den Schanz-Schrauben orientieren.
